# Redox-Sensitive Up-Regulation of eNOS by Purple Grape Juice in Endothelial Cells: Role of PI3-Kinase/Akt, p38 MAPK, JNK, FoxO1 and FoxO3a

**DOI:** 10.1371/journal.pone.0057883

**Published:** 2013-03-22

**Authors:** Mahmoud Alhosin, Eric Anselm, Sherzad Rashid, Jong Hun Kim, Socorro Vanesca Frota Madeira, Christian Bronner, Valérie B. Schini-Kerth

**Affiliations:** 1 CNRS UMR 7213 Laboratoire de Biophotonique et Pharmacologie, Université de Strasbourg, Faculté de Pharmacie, Illkirch, France; 2 Departamento de Fisiologia e Farmacologia, Universidade Federal do Ceará, Fortaleza, Brazil; Massachusetts Eye & Ear Infirmary, Harvard Medical School, United States of America

## Abstract

The vascular protective effect of grape-derived polyphenols has been attributable, in part, to their direct action on blood vessels by stimulating the endothelial formation of nitric oxide (NO). The aim of the present study was to determine whether Concord grape juice (CGJ), which contains high levels of polyphenols, stimulates the expression of endothelial NO synthase (eNOS) in porcine coronary artery endothelial cells and, if so, to determine the signaling pathway involved. CGJ dose- and time-dependently increased eNOS mRNA and protein levels and this effect is associated with an increased formation of NO in endothelial cells. The stimulatory effect of CGJ on eNOS mRNA is not associated with an increased eNOS mRNA stability and inhibited by antioxidants such as MnTMPyP, PEG-catalase, and catalase, and by wortmannin (an inhibitor of PI3-kinase), SB 203580 (an inhibitor of p38 MAPK), and SP 600125 (an inhibitor of JNK). Moreover, CGJ induced the formation of reactive oxygen species (ROS) in endothelial cells and this effect is inhibited by MnTMPyP, PEG-catalase, and catalase. The CGJ-induced the phosphorylation of p38 MAPK and JNK kinases is abolished by MnTMPyP. CGJ induced phosphorylation of transcription factors FoxO1 and FoxO3a, which regulate negatively eNOS expression, and this effect is prevented by MnTMPyP, PEG-catalase, wortmannin, SB203580 and SP600125. Moreover, chromatin immunoprecipitation assay indicated that the FoxO3a protein is associated with the eNOS promoter in control cells and that CGJ induced its dissociation. Thus, the present study indicates that CGJ up-regulates the expression of eNOS mRNA and protein leading to an increased formation of NO in endothelial cells. The stimulatory effect of CGJ is a redox-sensitive event involving PI3-kinase/Akt, p38 MAPK and JNK pathways, and the inactivation of the FoxO transcription factors, FoxO1 and FoxO3a, thereby preventing their repression of the eNOS gene.

## Introduction

Several epidemiological studies have suggested that regular intake of polyphenolic rich meals including vegetables, fruits and beverages such as red wine and green tea, is associated with beneficial effects on the cardiovascular system [1], [2], [3]. The protective effect of polyphenols on the cardiovascular system has been attributable, at least in part, to their ability to prevent oxidation of low-density lipoproteins [4], , platelet aggregation and adhesion [6], [7], and smooth muscle cell migration and proliferation [8], [9]. Moreover, vascular protection might also be due to the direct action of polyphenols on blood vessels by stimulating the formation of nitric oxide (NO), which is a potent vasodilator and inhibitor of platelet activation, in endothelial cells [3], [10], [11], [12]. Indeed, red wine polyphenols have been shown to cause the redox-sensitive activation of the PI3-kinase/Akt pathway leading to the phosphorylation of eNOS at Ser 1177 and the formation of NO [10], [12].

Grape-derived products such as red wine contain high levels of polyphenols, which are predominantly found in skins, seeds and stems. Besides red wines, grape juices, non-alcoholic beverages, are excellent alternative sources of grape-derived polyphenols. Previous studies have shown that ingestion of purple grape juice has protective effects on the vascular system by improving flow-mediated vasodilatation, platelet function and platelet-dependent inflammatory responses in patients with coronary artery disease [5], [13], [14], and by reducing blood pressure in moderately hypertensive patients [15]. In addition, consumption of purple grape juice increased serum antioxidant capacity and protected LDL against oxidation in healthy subjects [16]. In addition, we have shown that purple grape juice caused within seconds endothelium-dependent NO-mediated relaxations of coronary artery rings [12]. The signaling pathway leading to eNOS activation in response to grape juice is initiated by the intracellular formation of reactive oxygen species (ROS), in particular superoxide anions, which activate the Src/PI3-kinase/Akt pathway leading to the phosphorylation of eNOS at Ser 1177 [12].

Besides causing a rapid activation of eNOS, polyphenols might also induce a more sustained formation of NO by up-regulating the expression of eNOS in endothelial cells. Indeed, red wine, resveratrol and an artichoke leaf extracts caused a 2-fold up-regulation of eNOS mRNA and protein levels resulting in an increased formation of NO [17], [18], [19]. Since previous publications have shown that ROS especially hydrogen peroxide (H_2_O_2_) are able to induce the expression of eNOS [20], [21], the aim of the present study was to determine whether grape juice (CGJ) stimulates the expression of eNOS in coronary artery endothelial cells via a redox-sensitive mechanism and, if so, to determine the signaling pathway involved.

## Methods and Materials

### Chemicals

Superoxide dismutase (SOD), catalase, polyethyleneglycol-catalase (PEG-catalase), N^ω^-nitro-L-arginine (L-NA), SP 600125, actinomycin D and dihydroethidine were from Sigma (St. Louis, MO). Wortmannin, PD98059, SB203580 and the SOD mimetic Mn(III)tetrakis(1-methyl-4-pyridyl)porphyrin (MnTMPyP) were from Alexis Chemicals and PP2 (4-amino-5-(4-chlorophenyl)-7-(t-butyl)pyrazolo[3,4-d]pyrimidine) from Calbiochem. Concord grape juice (CGJ total phenolics: 2307 mg/l gallic acid equivalent; anthocyanins: 411 mg/l malvidin; proanthocyanidins: 509 mg/l catechin; potassium: 1460 mg/l) was provided by Welch Foods Inc. (Concord, MA, USA).

### Culture of Coronary Artery Endothelial Cells

Pig hearts were collected from the local slaughterhouse. Left circumflex coronary arteries were excised, cleaned of loose connective tissue and flushed with PBS without calcium to remove remaining blood. Thereafter, endothelial cells were isolated by collagenase treatment (type I, Worthington, 1 mg/ml for 12 minutes at 37°C), and cultured in culture dishes containing medium MCDB 131 (Invitrogen) and 15% fetal calf serum supplemented with penicillin (100 U/mL), streptomycin (100 U/mL), fungizone (250 µg/mL), and L-glutamine (2 mM) (all from Cambrex), and grown for 48–72 hours. All experiments were performed with confluent cultures of endothelial cells used at first passage. Cells were exposed to serum-free culture medium in the presence of 0.1% bovine serum albumin (QBiogene) for 6 hours prior to treatment and subsequent determination of the phosphorylation level of p38 MAPK, JNK, FoxO1 and FoxO3a.

### In situ Detection of NO

The formation of NO was assessed in endothelial cells using the fluorescent probe 4,5-diaminofluorescein-diacetate (DAF2-DA). They were exposed to serum-free culture medium in the presence of 0.1% bovine serum albumin for 6 hours prior to treatment. Endothelial cells were treated with CGJ (5.5 mg/l) for 24 hours and then they were exposed to DAF2-DA (1 µM) for 20 minutes at 37°C, in the absence or presence of N^ω^-nitro-L-arginine (L-NA; 1 mM). Thereafter, DAF2 fluorescence was determined by confocal microscope (1024 MRC; Bio-Rad, Hercules, CA) with a 10x epifluorescence objective (Nikon, Tokyo, Japan). After excitation at 488 nm with a Krypton/Argon laser, the emission signal was recorded with a Zeiss 565–610 nm filter. Images were analyzed using Photoshop software.

### Determination of the Cellular Formation of ROS

The oxidative fluorescent dye dihydroethidine was used to evaluate the *in situ* formation of ROS. Porcine coronary artery endothelial cells were cultured in Lab-Tek chamber slides (Thermo Fisher Scientific) until they reached 70–80% of confluence. To determine the nature of ROS, cells were incubated either with MnTMPyP (100 µM), SOD (500 U/ml), catalase (500 U/ml), or PEG-catalase (500 U/ml) for 30 minutes at 37°C before the addition of dihydroethidine (10 µM) for 30 minutes. Then, cells were challenged with or without CGJ (11 mg/l) for 30 minutes. Chamber slides were then washed three times with PBS, mounted in Vectashield and cover-slipped. Images were obtained with a Leica SP2 UV DM IRBE laser scanning confocal microscope. Quantification of staining levels was performed using FIJI GPL v2 software.

### Western Blot Analysis

After treatment, endothelial cells were washed twice with PBS and then lysed in extraction buffer (composition in mM: Tris/HCl 20 (pH 7.5; QBiogene), NaCl 150, Na_3_VO_4_ 1, sodium pyrophosphate 10, NaF 20, okadaic acid 0.01 (Sigma), a tablet of protease inhibitor (Roche) and 1% Triton X-100 (QBiogen)). Total proteins (25 µg) were separated on 6–10% SDS-polyacrylamide (Sigma) gels at 100 V for 2 hours. Separated proteins were transferred electrophoretically onto polyvinylidine difluoride membranes (Amersham) at 100 V for 120 minutes. Membranes were blocked with blocking buffer containing 5% bovine serum albumin, Tris-buffered saline solution (Biorad) and 0.1% Tween 20 (Sigma) (TBS-T) for 1 hour. For detection of phosphorylated proteins, membranes were incubated with the respective primary antibody (p–p38 MAPK Thr186/Tyr182, p-JNK Thr183/Tyr185, p-FoxO1 Thr24/FoxO3a Thr32) and for total eNOS, membranes were incubated with a primary antibody directed against eNOS (Cell Signaling Technology; dilution of 1∶1,000) overnight at 4°C. Detection of β-tubulin protein was used for normalization and quantification. After washing, membranes were incubated with the appropriate horseradish peroxidase-conjugated secondary antibody (diluted to 1∶20,000 for anti-mouse antibody and 1∶5,000 for anti-rabbit antibody, Cell Signaling Technology) at room temperature for 60 minutes. Prestained markers (Invitrogen) were used for molecular mass determinations. Immunoreactive bands were detected by enhanced chemiluminescence (Amersham).

### Real-Time PCR

Total RNA was isolated from endothelial cells using RNeasy Micro kit (Qiagen, Courtaboeuf, France). cDNA was synthesized from total RNA using iScript cDNA Synthesis kit (Bio-Rad, Marnesla-Coquette, France), and PCR amplification was performed using IQ SYBR Green Supermix (Bio-Rad). The specific primers were as follows: eNOS sense, 5′-AGCGGCTGCATGACATTGAG-3′, eNOS antisense, 5′-GTCGCCGCAGACAAACATGT-3′. The control housekeeping gene was porcine GAPDH. Relative quantitation was determined by standard 2^(–ΔΔCT)^ calculations.

### Chromatin Immunoprecipitation (ChIP) assay

Coronary artery endothelial cells (approximately 1.5×10^7^) were cross-linked for 10 minutes at room temperature by adding 37% formaldehyde to the culture medium. The fixed cells were lysed and the chromatin was isolated using ChIP-IT^TM^ Express Enzymatic (Active Motif, Carlsbad, California, USA). For ChIP, sheared chromatin was incubated with rabbit polyclonal to FoxO3a-ChIP Grade (Abcam, Cambridge, UK). The following steps were performed according to the manufacture's instructions. The isolated precipitated DNA was analysed by PCR amplification of approximately 215-bp fragment of the human eNOS promoter (forward, 5′-CGGAGCAGGTGATAGAAGCTAGG-3′ and reverse, 5′-GCTTCCCTGGAGTCTTGTGTAAGG-3′.

### Statistical Analysis

Values are expressed as means ± SEM. Statistical evaluation was performed with Student's *t* test for paired data or ANOVA followed by Fischer's protected least significant difference test where appropriate. Values of *P*<0.05 were considered statistically significant.

## Results

### Concord grape juice up-regulates eNOS mRNA level in endothelial cells

**Figure 1:**
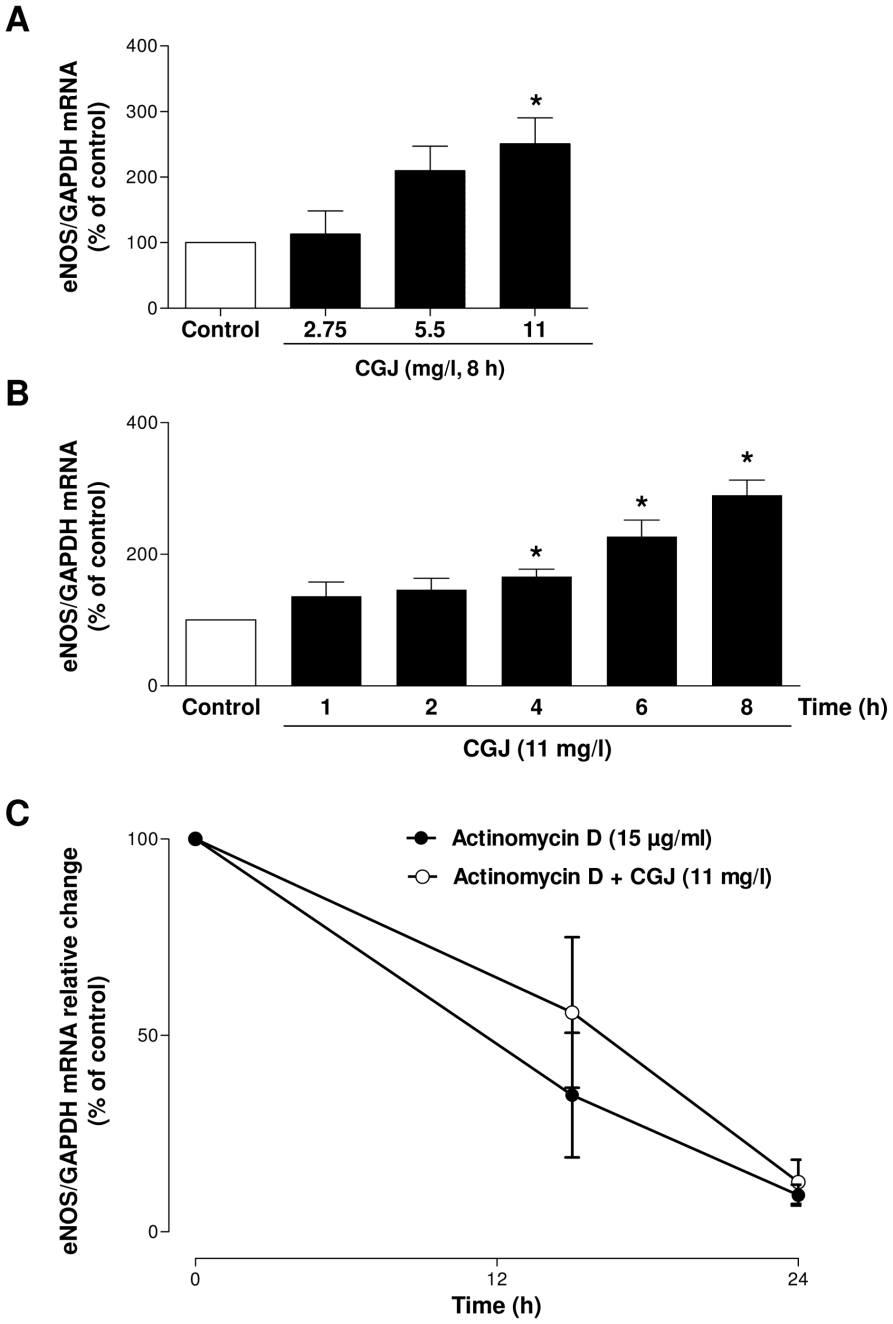
CGJ up-regulates eNOS mRNA expression in coronary artery endothelial cells. Endothelial cells were incubated either with different concentrations of CGJ for 8 hours (A) or CGJ (11 mg/l) in a time-dependent manner until 8 hours (B). Then total mRNA was extracted and transcripted into cDNA. The level of eNOS mRNA was determined using RT-PCR analysis. n = 6 different experiments. CGJ did not affect eNOS mRNA stability (C). Actinomycin (15 µg/mL), an inhibitor of transcription, was added to endothelial cells in the presence or absence of CGJ (11 mg/l). n = 4 different experiments *P<0.05 versus control.

To determine the effect of long-term treatment of endothelial cells with CGJ, eNOS mRNA levels were determined as a function of concentration and time using RT-PCR As shown in Fig. 1A, treating endothelial cells with CGJ for 8 hours induced a concentration-dependent increase of the eNOS mRNA level, which reached significance at 11 mg/l. Thereafter, this concentration was used to study the time course of the stimulatory effect of CGJ on eNOS mRNA level (Fig. 1B) CGJ induced a time-dependent increase of the eNOS mRNA level, which reached significance at 4 hours and, thereafter, it increased steadily up to, at least, 8 hours. To determine whether this effect is due to an increased stability of eNOS mRNA, cells were exposed to actinomycin D, an inhibitor of transcription, in the absence and presence of CGJ for 15 and 24 hours. CGJ did not affect the time-dependent decrease of eNOS mRNA indicating that the stimulatory effect of CGJ is not due to an increased stability of eNOS mRNA (Fig. 1C).

### Concord grape juice up-regulates eNOS protein leading to an enhanced formation of NO in endothelial cells

**Figure 2:**
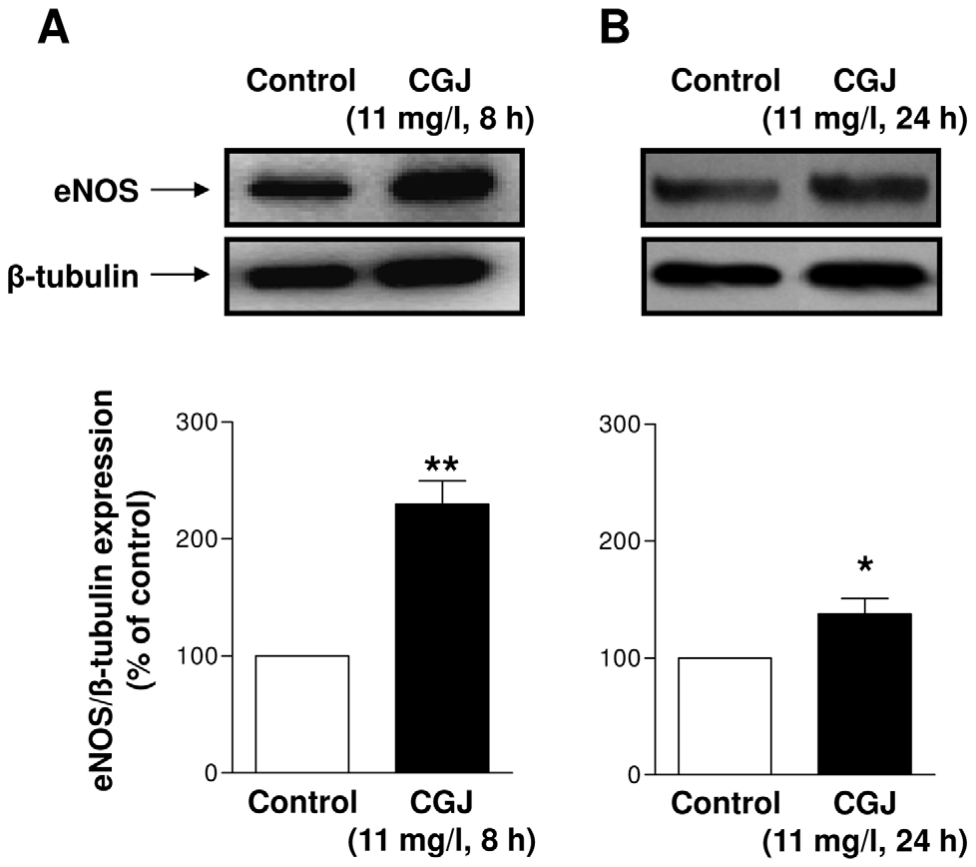
CGJ up-regulates eNOS protein level in endothelial cells. Endothelial cells were stimulated with CGJ for 8 (A) and 24 hours (B) and then the proteins were extracted. The level of eNOS protein was determined by Western blot analysis (top) and by densitometric analysis (bottom). n = 9 different experiments. *P<0.05 versus control.

**Figure 3:**
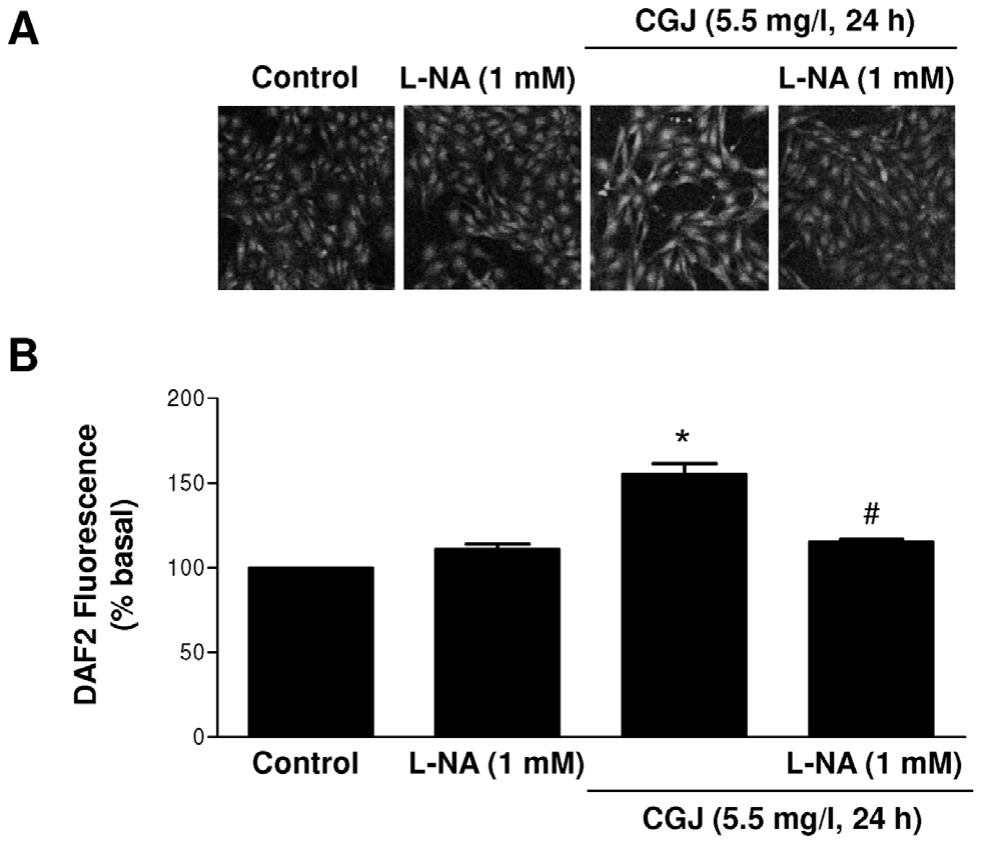
CGJ stimulates the endothelial formation NO. NO formation was assessed using the fluorescent NO-sensitive probe diaminofluorescein-2 diacetate (DAF-2DA) by confocal microscope. n = 3 different experiments. *P<0.05 versus control.^ #^ *P*<0.05 versus CGJ treatment.

Next, Western blot analysis was performed to verify that the increased eNOS mRNA level induced by CGJ leads to an increased eNOS protein level. After an 8-hour treatment period, CGJ (11 mg/l) significantly increased the eNOS protein level compared to control cells, and this effect persisted up to 24 hours (Fig. 2). In order to determine that the CGJ-induced expression of eNOS is associated with an enhanced formation of NO, endothelial cells were exposed to a fluorescent probe known to detect NO, DAF2-DA. As shown in Fig. 3, the fluorescence signal was significantly higher after a 24-hour treatment period of endothelial cells with CGJ. Pre-treatment of endothelial cells with the competitive inhibitor of eNOS, L-NA, prevented the stimulatory effect of CGJ (Fig. 3). These data indicate that CGJ increased the eNOS-derived NO formation in endothelial cells.

### CGJ induces a redox-sensitive expression of eNOS mRNA in endothelial cells

**Figure 4:**
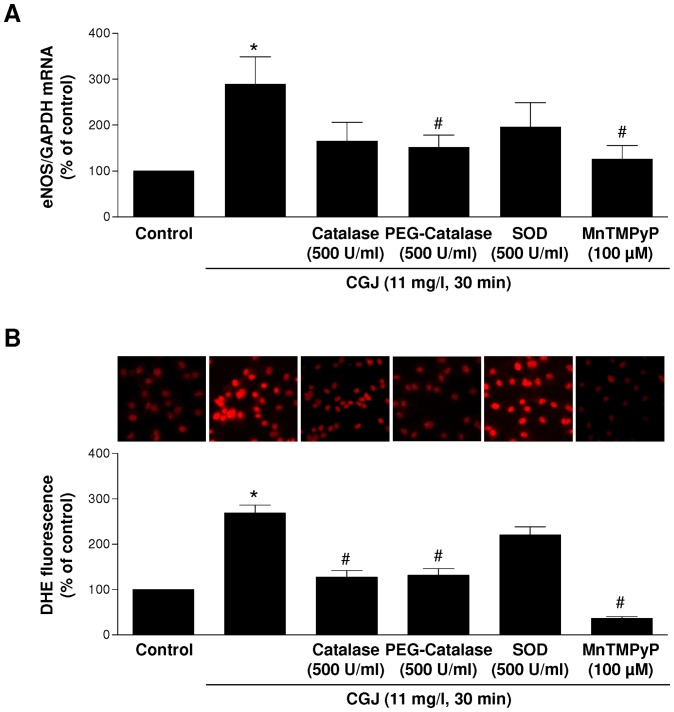
CGJ induces a redox-sensitive up-regulation of eNOS mRNA. Endothelial cells were exposed to either solvent or CGJ for 6 hours at 37°C. Catalase (500 U/ml), PEG-catalase (500 U/ml), SOD (500 U/ml) and MnTMPyP (100 µM), were added to cells 30 minutes before the addition of CGJ and then total mRNA was extracted. RT-PCR analysis was performed to detect the level of mRNA coding for eNOS (A). n = 6 different experiments. CGJ induces the formation of ROS in endothelial cells (B). *P<0.05 versus control. ^#^ *P*<0.05 versus CGJ treatment.

Previous studies have shown that ROS are able to stimulate the expression of eNOS in endothelial cells [20], [21]. Moreover, we have previously shown that CGJ induces the formation of ROS in coronary artery endothelial cells leading acutely to eNOS activation [12]. Therefore, we performed experiments to determine the role of ROS in the up-regulation of eNOS induced by CGJ. Modulators of ROS strongly inhibited the expression of eNOS induced by CGJ. Indeed as shown in Fig. 4A, membrane-permeant analogs of either SOD (MnTMPyP) or catalase (PEG-catalase) significantly prevented the increased eNOS mRNA level induced by CGJ. Although native SOD and catalase reduced CGJ-induced eNOS expression, this effect did not reach statistical significance (Fig. 4A). In addition, MnTMPyP or PEG-catalase alone affected little eNOS mRNA levels in control endothelial cells (eNOS mRNA level were 104.9±14.1% and 107.3±18.5% compared to control cells, n = 3). Thus, these findings indicate a major role of intracellular ROS and especially superoxide anions and H_2_O_2_ in the signaling pathway leading to the expression of eNOS in response to CGJ.

**Figure 5:**
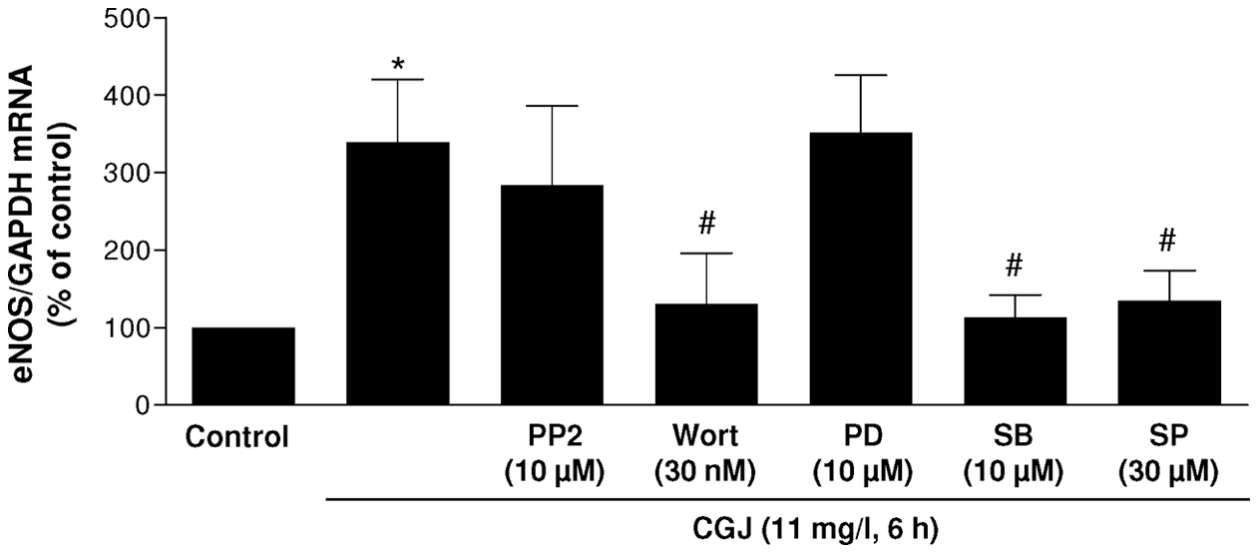
PI3-kinase, p38 MAPK and JNK mediate the redox-sensitive CGJ-increased expression of eNOS. Endothelial cells were exposed to CGJ for 6 hours at 37°C with either solvent, PP2 (10 µM, an inhibitor of Src), wortmannin (30 nM, an inhibitor of PI3K), PD 098059 (10 µM, an inhibitor of ERK 1/2), SB 203580 (10 µM, an inhibitor of p38 MAPK) and SP 600125 (30 µM, an inhibitor of JNK) for 30 minutes before the addition of CGJ and then total mRNA was extracted. RT-PCR analysis was performed to detect the level of mRNA coding for eNOS. n = 5 to 10 different experiments. *P<0.05 versus control. ^#^ *P*<0.05 versus CGJ treatment.

Direct evidence that CGJ stimulates the formation of ROS in endothelial cells was obtained using the redox-sensitive probe DHE (Fig. 4B). CGJ increased about two-fold the DHE fluorescence signal, and this effect was abolished by MnTMPyP, and significantly reduced by catalase and PEG-catalase, and not affected by SOD (Fig. 4B). Since ROS are well-known to activate redox-sensitive kinases in endothelial cells to induce biological responses such as cell growth, survival and apoptosis [22], experiments were performed to determine the role of Src-kinase using PP2, PI3-kinase using wortmannin, ERK1/2 (Extracellular signal-regulated kinase 1 and 2) using PD 098059, p38 MAPK using SB 203580, and JNK using SP 600125. As shown in Fig. 5, wortmannin, SB 203580 and SP 600125 significantly prevented the CGJ-induced expression of eNOS mRNA whereas PP2 and PD 098059 were without effect. In addition, the inhibitors alone affected little the basal eNOS mRNA expression level in endothelial cells (values were 94.2±10.2% and 85.0±2.9% for wortmannin and SP 600125, respectively, n = 3). Thus, these findings suggest a key role of PI3 kinase, p38 MAPK and JNK in the signal transduction pathway leading to eNOS expression in response to CGJ.

### CGJ causes the redox-sensitive activation of p38 MAPK and JNK

**Figure 6:**
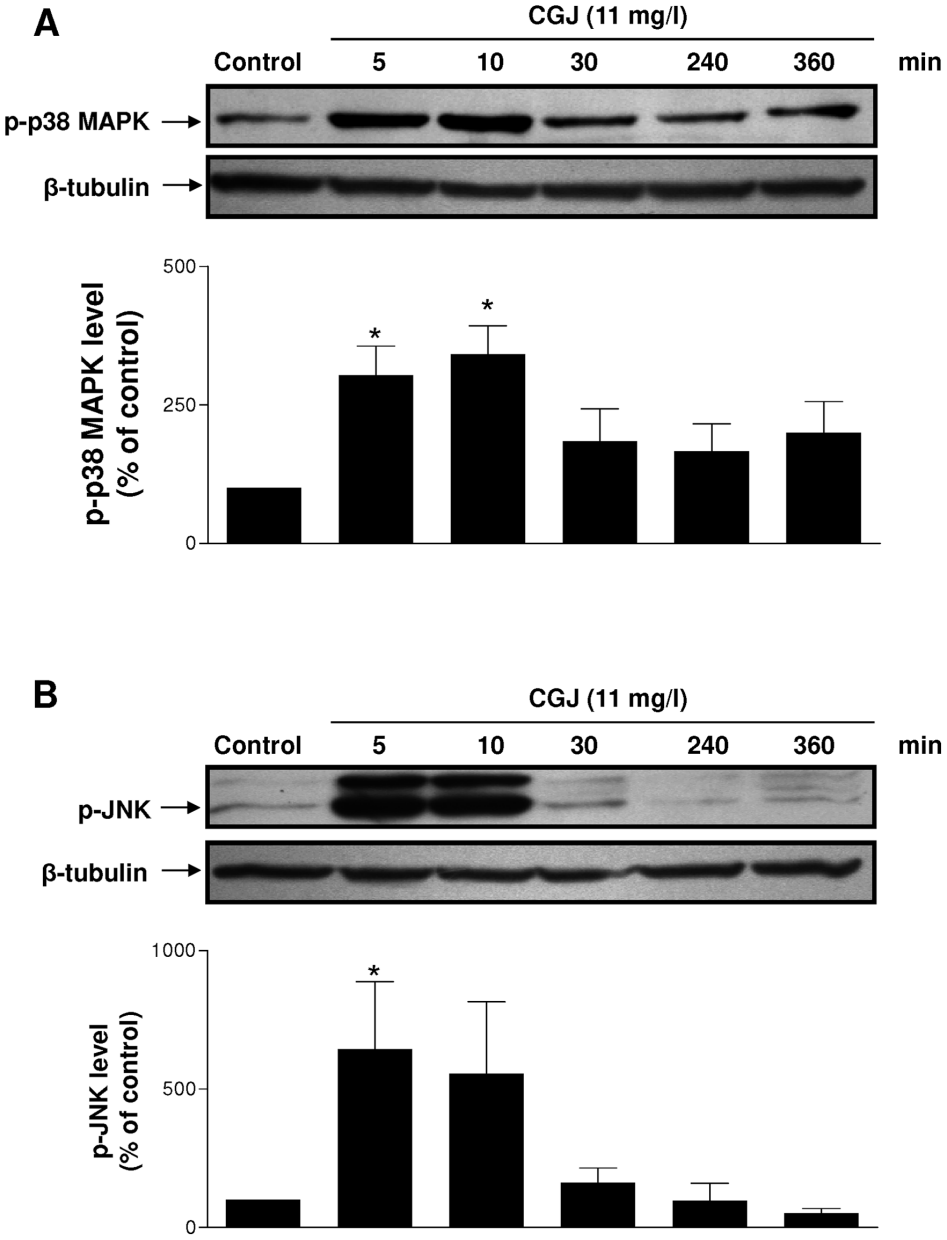
CGJ causes a time-dependent phosphorylation of p38 MAPK at Thr186/Tyr182 and JNK at Thr183/Tyr185 in endothelial cells. Cells were exposed to CGJ for the indicated times at 37°C. Thereafter, the level of p–p38 MAPK (A) and p-JNK (B) was determined by Western blot analysis. Top, representative immunoblots, and bottom, corresponding cumulative data. n = 5 different experiments. * *P*<0.05 versus control.

**Figure 7:**
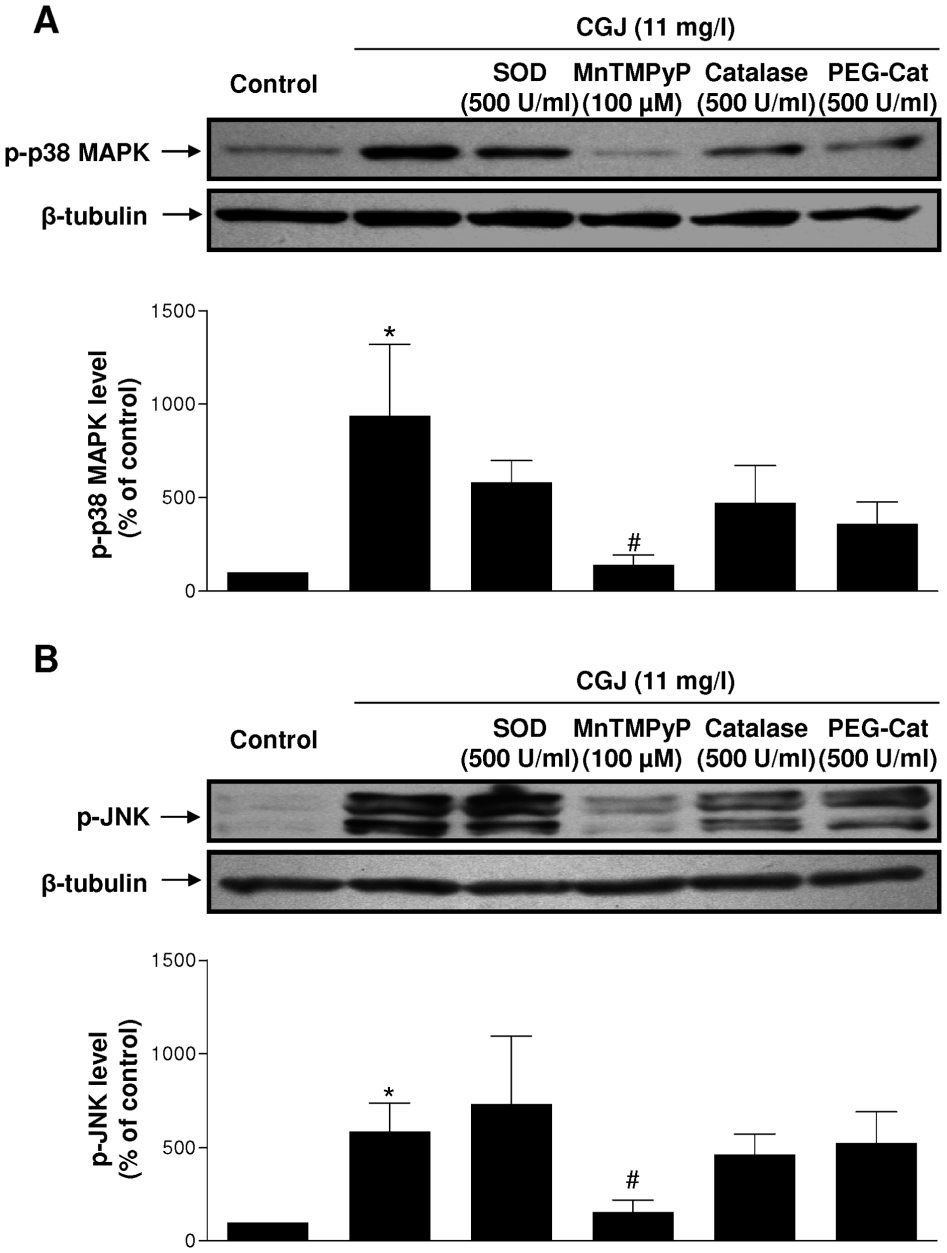
Role of reactive oxygen species in the CGJ-induced phosphorylation of p38 MAPK and JNK in endothelial cells. Cells were incubated either with solvent, SOD (500 U/ml), MnTMPyP (100 µM), catalase (500 U/m) and PEG-catalase (500 U/ml) for 30 minutes before the addition of CGJ for 5 minutes. The level of p–p38 MAPK (A) and p-JNK (B) was determined by Western blot analysis. Top, representative immunoblots, and bottom, corresponding cumulative data. n = 5 to 6 different experiments. * *P*<0.05 versus control. ^#^ *P*<0.05 versus CGJ treatment.

Unstimulated endothelial cells had either no or only a low level of p–p38 MAPK and p-JNK (Fig. 6 A,B). CGJ increased within 5 minutes signals of p–p38 MAPK and p-JNK, which reached a peak value within 5 to 10 minutes and then returned to baseline at 30 minutes. CGJ-induced phophorylation of p38 MAPK and JNK was abolished by MnTMPyP and not significantly reduced by native SOD, PEG-catalase and native catalase (Fig. 7 A,B). These data indicate that ROS, especially superoxide anions, act as intracellular upstream mediators of p38 MAPK and JNK leading to eNOS expression in response to CGJ.

### CGJ induced the inactivation of FoxO1 and FoxO3a

**Figure 8:**
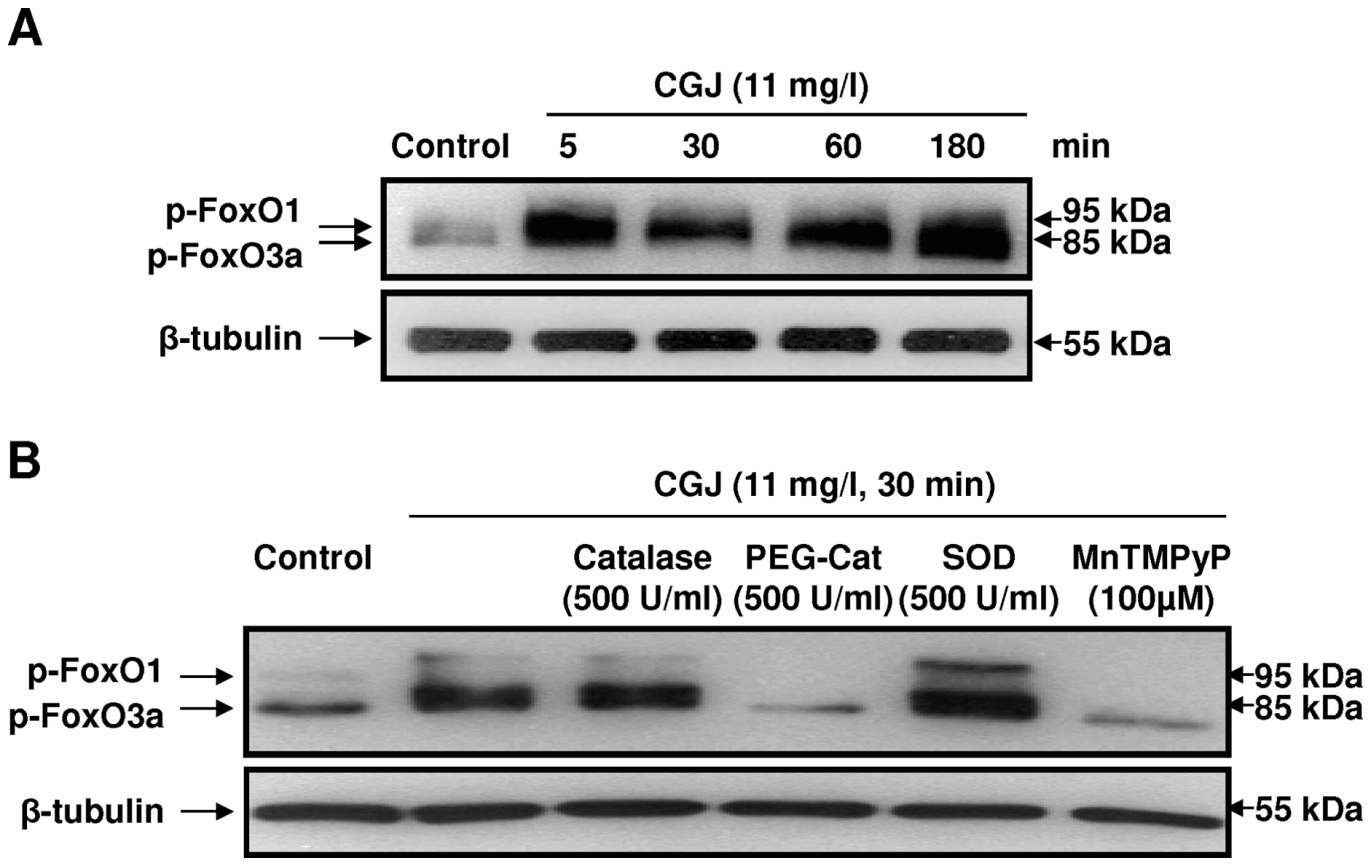
CGJ induces a redox-sensitive phosphorylation of FoxO1 and FoxO3a. Endothelial cells were exposed to CGJ for the indicated times at 37°C. The level of p-FoxO1 and p-FoxO3a was determined by Western blot analysis (A). Role of ROS in the CGJ-induced phosphorylation of FoxO1 and FoxO3a in endothelial cells (B). Cells were incubated either with solvent, SOD (500 U/ml), MnTMPyP (100 µM), catalase (500 U/ml) or PEG-catalase (500 U/ml) for 30 minutes before the addition of CGJ for 30 minutes.

**Figure 9:**
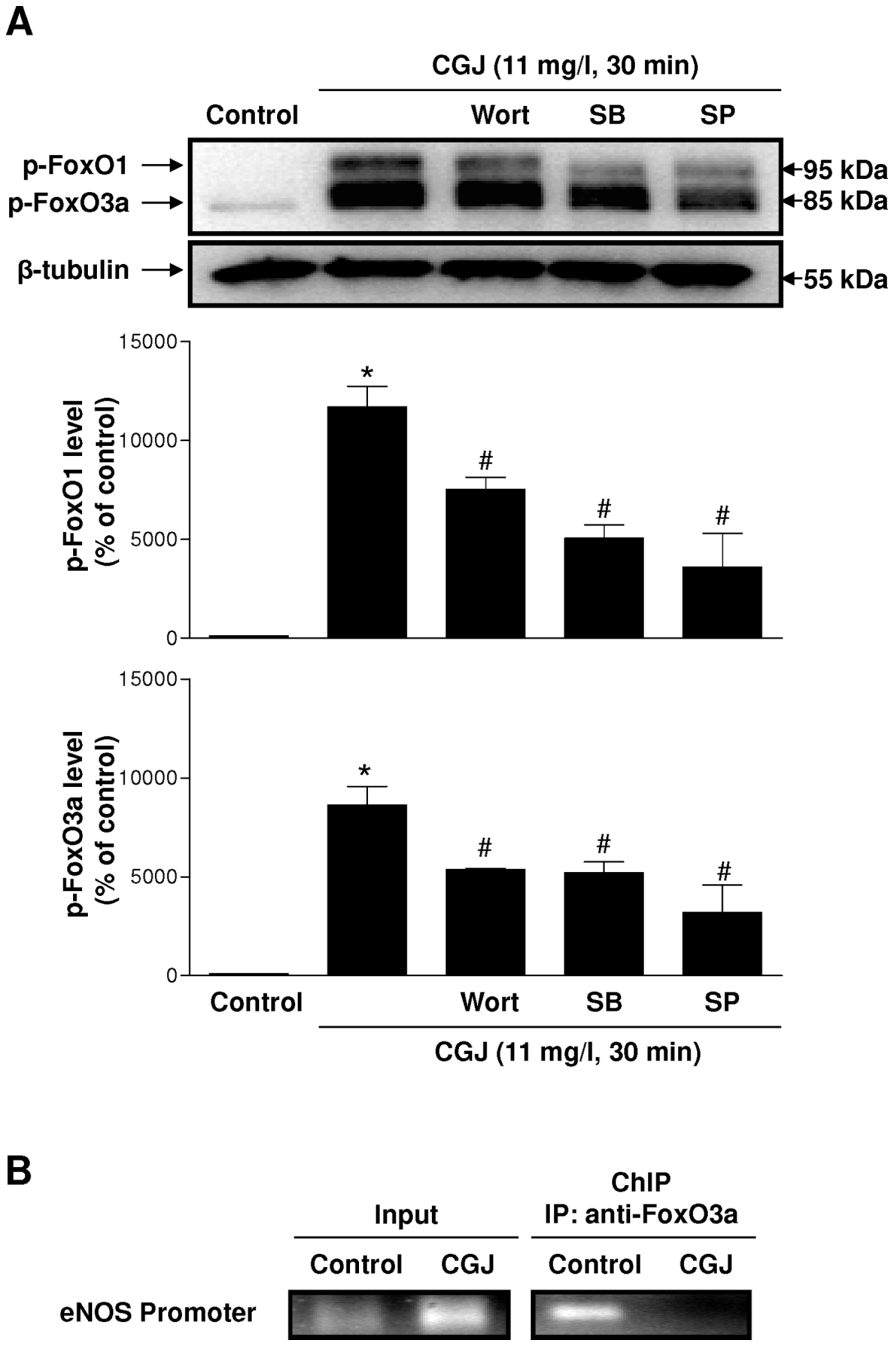
PI3-kinase, p38 MAPK and JNK mediate the redox-sensitive CGJ-induced phosphorylation of FoxO1 and FoxO3a leading to the FoxO3a dissociation from the eNOS gene promoter. Endothelial cells were incubated with wortmannin (30 nM), SB 203580 (10 µM) and SP 600125 (30 µM) for 30 minutes before the addition of CGJ for 30 minutes. The level of p-FoxO1 and p-FoxO3a protein (A) was determined by Western blot analysis (top) and by densitometric analysis (bottom). n = 4 different experiments. *P<0.05 versus control, ^#^ *P*<0.05 versus CGJ treatment. Endothelial cells were exposed to CGJ for 30 minutes and then chromatin-bound DNA was immunoprecipitated with an antibody against FoxO3a (B). Immunoprecipitated DNA was analyzed by PCR using primers to amplify a fragment of the human eNOS promoter as mentioned in methods and materials. n = 2 different experiments.

It has been shown that activation of the PI3-kinase pathway leads to an Akt-dependent inactivation of FoxO transcription factors resulting in a reduced DNA binding such as to the eNOS promoter [23], [24], [25]. To determine whether CGJ inactivates FoxO transcription factors, we studied the effect of CGJ on the phosphorylation of FoxO1 and FoxO3a. Exposure the endothelial cells to CGJ induced phosphorylation of the transcription factors FoxO1 and FoxO3a at 5 minutes and this effect persisted at least until three hours (Fig. 8A). Both MnTMPyP and PEG-catalase prevented the phosphorylation of FoxO1 and FoxO3a induced by CGJ (Fig. 8B) whereas native SOD and catalase had only minor effects (Fig. 8B). In addition, the CGJ-induced phosphorylation of FoxO1 and FoxO3a was significantly prevented by wortmannin, SB 203580 and SP 600125 (Fig. 9A). Chromatin immunoprecipitation (ChIP) assay showed that FoxO3a binds to the eNOS promoter and that CGJ targeted this interaction leading to FoxO3a dissociation from the eNOS promoter (Fig. 9B). Thus, these findings indicate an important role of intracellular ROS, p38 MAPK, JNK and PI3-kinase in the signal transduction pathway leading to phosphorylation of FoxO1 and FoxO3a in response to CGJ. Moreover they further support a key role of FoxO3a phosphorylation in the CGJ-induced eNOS gene activation.

## Discussion

The present findings indicate that CGJ stimulates the expression of eNOS both at the mRNA and protein level and that this effect is associated with a sustained formation of NO in endothelial cells. They further indicate that the stimulatory effect of CGJ on eNOS expression is initiated by a moderate pro-oxidant event involving superoxide anions and hydrogen peroxide, which regulate eNOS expression through activation of several kinases including PI-3-kinase, p38 MAPK and JNK. The PI3-kinase, p38 MAPK and the JNK pathways are involved in the phosphorylation of the transcription factors FoxO1 and FoxO3a thereby reducing their repressor effect on the expression of the eNOS gene.

Chronic intake of grape-derived polyphenols has been shown to induce vasoprotective effects in both humans and animals. Indeed, intake of wine, red wine without alcohol and purple grape juice improved flow-mediated vasodilatation of the brachial artery in healthy subjects and in subjects with coronary artery diseases [5], [26], [27]. Chronic intake of grape-derived polyphenols also prevented hypertension and improved endothelial dysfunction in several experimental models of hypertension [28], [29], [30]. The beneficial effects of grape-derived polyphenols involve, at least in part, their ability to enhance acutely the endothelial formation of NO and endothelium-derived hyperpolarizing factor, two major vasoprotecting factors [31], [32], [33], [34]. The grape-derived polyphenols-induced endothelial formation of NO is mediated by the phosphorylation of Ser 1177 of eNOS via the Src kinase/PI3-kinase/Akt pathway [12], [31]. Surprisingly, reactive oxygen species including superoxide anions and hydrogen peroxide act as upstream mediators of the Src kinase/PI3-kinase/Akt pathway [12], [31], [35]. Although the endothelial source of reactive oxygen species remains to be determined, the redox-sensitive NO- and EDHF (endothelium-derived hyperpolarizing factor)-mediated relaxations to grape-derived polyphenols are not affected by pharmacological inhibitors of the mitochondrial respiration chain, xanthine oxidase, and cytochromes P450, and NO-mediated relaxations persisted in NADPH oxidase gp91phox knockout mice ruling out these potential sources [10], [36]. Alternatively, the polyphenolic structure itself might provide the oxidative activator signal since the structure can undergo redox cycling leading to the formation of superoxide anions [37]. Besides grape-derived polyphenols, a redox-sensitive pathway involving the Src kinase/PI3-kinase/Akt pathway mediates also activation of eNOS in response to the major green tea polyphenol epigallocatechin-3-gallate [35], [38].

In addition to acutely stimulating the endothelial formation of NO, the vasoprotective effect of grape-derived polyphenols may also involve their ability to increase the expression level of eNOS mRNA and protein with a subsequent sustained formation of NO [17], [18], [19], [39], [40]. The present findings indicate that the CGJ induced an up-regulation of eNOS mRNA already after 4 hours and that this effect results in a 1.5-fold increased formation of NO after a 24 hour-incubation period. The stimulatory effect of CGJ is not due to an increased stability of eNOS mRNA.

Previous studies have indicated that red wine and resveratrol increased the activity of the eNOS promoter and also stabilized to some extent eNOS mRNA [18], [19], [39]. Altogether, these findings indicate that grape-derived polyphenols-induced up-regulation of eNOS expression involves transcriptional and possibly also post-transcriptional mechanisms. The present investigations further indicate that the stimulatory effect of grape-derived polyphenols on eNOS expression is critically dependent on a redox-sensitive event. Indeed, the CGJ-induced expression of eNOS mRNA is associated with the formation of cellular ROS and it is markedly reduced by membrane permeant analogs of superoxide dismutase and catalase indicating a key role of intracellular superoxide anions and hydrogen peroxide. Moreover, direct evidence that grape-derived polyphenols stimulate the intracellular formation of superoxide anions and hydrogen peroxide has also been obtained previously in both native and cultured endothelial cells but not in the vascular smooth muscle using redox-sensitive fluorescent probes [36], [41]. Previous studies have also shown that hydrogen peroxide caused a time- and concentration-dependent up-regulation of eNOS in endothelial cells through both transcriptional and post-transcriptional mechanisms [21]. Altogether, the present findings in conjunction with those previous ones indicate that both the acute activation of eNOS and its subsequent increased expression in response to grape-derived polyphenols are controlled by a pro-oxidant event in endothelial cells involving intracellular superoxide anions and hydrogen peroxide. A role for intracellular hydrogen peroxide has also been suggested in oscillatory shear stress-induced up-regulation of eNOS mRNA and NO formation in endothelial cells since both of these responses were markedly reduced by PEG-catalase [42].

The fact that reactive oxygen species act as important endogenous signaling molecules modulating gene expression in endothelial cells through activation of redox-sensitive intracellular targets such as protein kinases including Src kinase, PI3-kinase, ERK1/2, p38 MAPK and JNK prompted experiments to determine their role in the CGJ-induced expression of eNOS [21], [43], [44], [45]. The present findings indicate that the stimulatory effect of CGJ is abolished by a selective inhibitor of either PI3-kinase, p38 MAPK or JNK whereas inhibition of Src kinase or ERK1/2 was without effect. Moreover, CGJ induced within 5 minutes the phosphorylation of p38 MAPK and JNK, both of these responses were transient and returned to baseline within 30 minutes. Intracellular superoxide anions have an important role in the CGJ-induced phosphorylation of p38 MAPK and JNK since both responses are abolished by MnTMPyP and not significantly affected by native superoxide dismutase, catalase and PEG-catalase. In addition, we have previously shown that CGJ also causes within minutes the PI3-kinase-dependent phosphorylation of Akt and that this effect is dependent on intracellular superoxide anions and hydrogen peroxide [12]. Thus, CGJ induces expression of eNOS in endothelial cells resulting in a sustained formation of NO through the redox-sensitive activation of several intracellular signaling pathways involving PI3-kinase/Akt, p38 MAPK and JNK.

Previous studies have indicated that the eNOS promoter region contains putative binding sites for redox-sensitive transcription factors, including FoxO1 and FoxO3a, activator protein-1 (AP-1), Sp1, and antioxidant-responsive elements [23], [46], [47], [48]. Indeed, FoxO1 and FoxO3a have been shown to bind to the eNOS gene promoter and to repress eNOS expression [23]. The present findings indicate that the FoxO3a protein is associated with the eNOS promoter in control endothelial cells and that CGJ induced its dissociation most likely following the phosphorylation of FoxO3a leading to its exclusion from the nucleus into the cytoplasm. Thus, the CGJ-induced phosphorylation of FoxO1 and FoxO3a appears to be an important event leading to eNOS expression.

In addition, recent findings suggest that the stimulatory effect of grape-derived polyphenols on eNOS expression is also observed *in vivo* since intake of red wine polyphenols in the drinking water (150 mg/kg/day) during 3 weeks is associated with a significant 1.6-fold up-regulation of the eNOS protein level in the rat aorta [49].

In conclusion, the present findings indicate that CGJ caused an up-regulation of eNOS resulting in a sustained formation of NO and that this effect is critically dependent on the intracellular formation of superoxide anions and hydrogen peroxide. They further indicate that the stimulatory effect on eNOS expression involves several redox-sensitive kinases including PI3-kinase, p38 MAPK, JNK and the transcription factors FoxO1 and FoxO3a. Thus, the dual ability of grape-derived polyphenols to acutely enhance the endothelial formation of NO by changing the phosphorylation level of eNOS and to cause a more sustained endothelial formation of NO following up-regulation of the eNOS protein might contribute to explain its protective effect on the vascular system.
